# A large-scale mosquito larviciding in Tanga Region, Tanzania, reduced mosquito densities to varying degrees across malaria transmission risk strata

**DOI:** 10.1038/s41598-025-26675-w

**Published:** 2025-11-27

**Authors:** Tegemeo Gavana, Denis Kailembo, Elizabeth Kasagama, Nasoro S. Lilolime, Tunu G. Mwamlima, Charles Dismas Mwalimu, Jubilate Bernard, Best Yoram, Stella Kajange, Samwel Lazaro, Noela Kisoka, Prosper Chaki, Christian Lengeler

**Affiliations:** 1 Environmental Health and Ecological Sciences, Ifakara Health Institute, Dar es Salaam, United Republic of Tanzania; 2 Swiss Tropical and Public Health Institute, Kreuzstrasse 2, Allschwil, 4123 Basel, Switzerland; 3 University of Basel, Petersplatz 1, Basel, Switzerland; 4 Ifakara Health Institute, Bagamoyo, United Republic of Tanzania; 5 National Malaria Control Programme, Ministry of Health, Dodoma, Tanzania; 6 Regional Administration and Local Government, President Office, Government City, Mtumba TAMISEMI street, Dodoma, Tanzania

**Keywords:** Vector control, Biolarviciding, *Bacillus sphaericus*, *Bacillus thuringiensis israelensis*, Bactivec^®^, Griselesf^®^, Malaria, Entomology

## Abstract

In 2019, the Government of Tanzania endorsed a nationwide scale-up of mosquito larviciding. Prior to full implementation, a pilot project was conducted in the Tanga Region from June 2022 to April 2024. The intervention targeted three councils representing high, moderate, and low malaria epidemiological risk strata. Six rounds of larvicide application were conducted, each lasting eight weeks and scheduled according to local rainfall patterns. All mosquito breeding habitats identified by trained community members were treated using *Bacillus thuringiensis israelensis* and *Bacillus sphaericus*. Each intervention council was paired with a control council, and longitudinal entomological monitoring was conducted in 120 villages (60 intervention, 60 control). Larviciding was generally associated with lower densities of late-stage *Anopheles* larvae across strata, with Incidence Rate Ratios (IRRs) of 0.20 (95% CI: 0.12–0.35) in high-risk, 0.51 (95% CI: 0.26–1.01) in moderate-risk, and 0.40 (95% CI: 0.24–0.68) in low-risk areas. Adult *Anopheles gambiae* sensu lato densities were also reduced in moderate- and low-risk strata, while no significant reductions were observed in *Anopheles funestus* populations. These findings suggest that larviciding can reduce mosquito densities in varied ecological settings, though effectiveness may vary by species and transmission context. Optimizing implementation strategies may enhance the entomological impact.

**Supplementary Information:**

The online version contains supplementary material available at 10.1038/s41598-025-26675-w.

## Introduction

Vector control interventions, particularly Insecticide-Treated mosquito Nets (ITNs) and Indoor Residual Spraying (IRS), have made a substantial contribution to the reduction of malaria burden globally^1^. Their massive roll-out from 2004 onwards is estimated to have prevented 2.1 billion malaria cases and 7·6 million deaths in sub-Saharan Africa, and 60% of the prevented deaths are attributed to the effectiveness of the ITNs alone^2^.

Despite the continued use of the two interventions, malaria control progress has stagnated since 2016, with some countries experiencing an increase in malaria cases and deaths. According to the 2017 World Malaria Report, 216 million malaria cases and 445,000 deaths related to malaria occurred worldwide^3^. By 2023, the number of cases had increased to 249 million, with 608,000 deaths globally^4^. The most recent report in 2024 showed that the malaria burden has remained largely unchanged, with 263 million cases and 597,000 deaths worldwide^1^. Malaria is more prevalent in the World Health Organization (WHO) African Region, contributing about 94% of the cases and 95% deaths^1,4^.

This persistent stagnation, as reported in recent years^1,4–10^, underscores the need for additional interventions to accelerate malaria control efforts toward elimination, especially in Africa^1,8,9^. The WHO recommends maintaining sufficient coverage of ITNs and/or IRS as the core malaria vector control interventions, while also integrating secondary interventions such as larviciding in areas that are suitable, and where optimal ITNs and IRS coverage has been attained but malaria transmission continues^5,11–13^.

Larviciding refers to the application of chemical or biological agents called larvicides in water bodies or water containers, to reduce the number of aquatic-stage mosquitoes^13,14^. The theory of change behind larviciding is that targeting mosquitoes in their aquatic stages reduces the number of larvae developing into adult mosquitoes, thereby reducing the number that would bite humans and infect them with malaria or other mosquito-borne infections^14^.

The current position of the WHO on Larval Source Management (LSM) is that larviciding should be implemented as a supplementary intervention in areas where breeding habitats are Few, Fixed and Findable (the 3Fs), making the intervention mainly suitable in urban settings and semi-arid areas^13,15^. This recommendation is primarily based on operational challenges in identifying large number of breeding sites and cost-effectiveness concerns in areas with many scattered larval habitats^16–20^. However, advancements in technologies^16–30^, and the increasing relevance of vector species such as *Aedes aegypti*^31–33^ and *Anopheles stephensi*^34–41^, which are difficult to control using only ITNs or IRS, have stimulated discussion about re-visiting the value of larviciding.

Tanzania remains on the list of high malaria burden countries in the world, contributing about 5% of all the global malaria deaths^42^. The National Malaria Control Program (NMCP) has endorsed the countrywide implementation of larviciding in its National Malaria Strategic Plan 2021–2025^43^ and the National Strategy for Vector Control 2019–2024^44^. In order to inform the national scaling-up, the NMCP and its partners implemented a pilot larviciding intervention in Tanga Region to generate evidence on (i) the feasibility of the intervention, (ii) its cost, and (iii) its entomological and epidemiological impact.

The pilot larviciding project in Tanga Region was implemented in three councils: Handeni District Council (DC), Tanga City Council (CC) and Lushoto DC. The three councils were selected pragmatically by the NMCP and partners to represent different malaria risk strata according to the National Malaria Strategic Plan: high risk (Handeni DC), moderate (Tanga CC) and low (Lushoto) while taking into account rural setting (Handeni DC and Lushoto DC) and urban settings (Tanga CC)^45–49^.

The project was conducted from June 2022 to April 2024. Over this period, six rounds of larvicide application, each lasting eight weeks, were conducted. The timing of these rounds was guided by local rainfall patterns, with applications scheduled during periods of no or minimal rainfall. In each round, all mosquito breeding habitats identified by community own resource personnel (CORPs) were treated using *Bacillus thuringiensis var. israelensis* and *Bacillus sphaericus* biolarvicides. This paper presents results of the entomological evaluation conducted to estimate the impact of larviciding on larval and adult mosquito populations.

## Methods

### Study setting

**Fig. 1:**
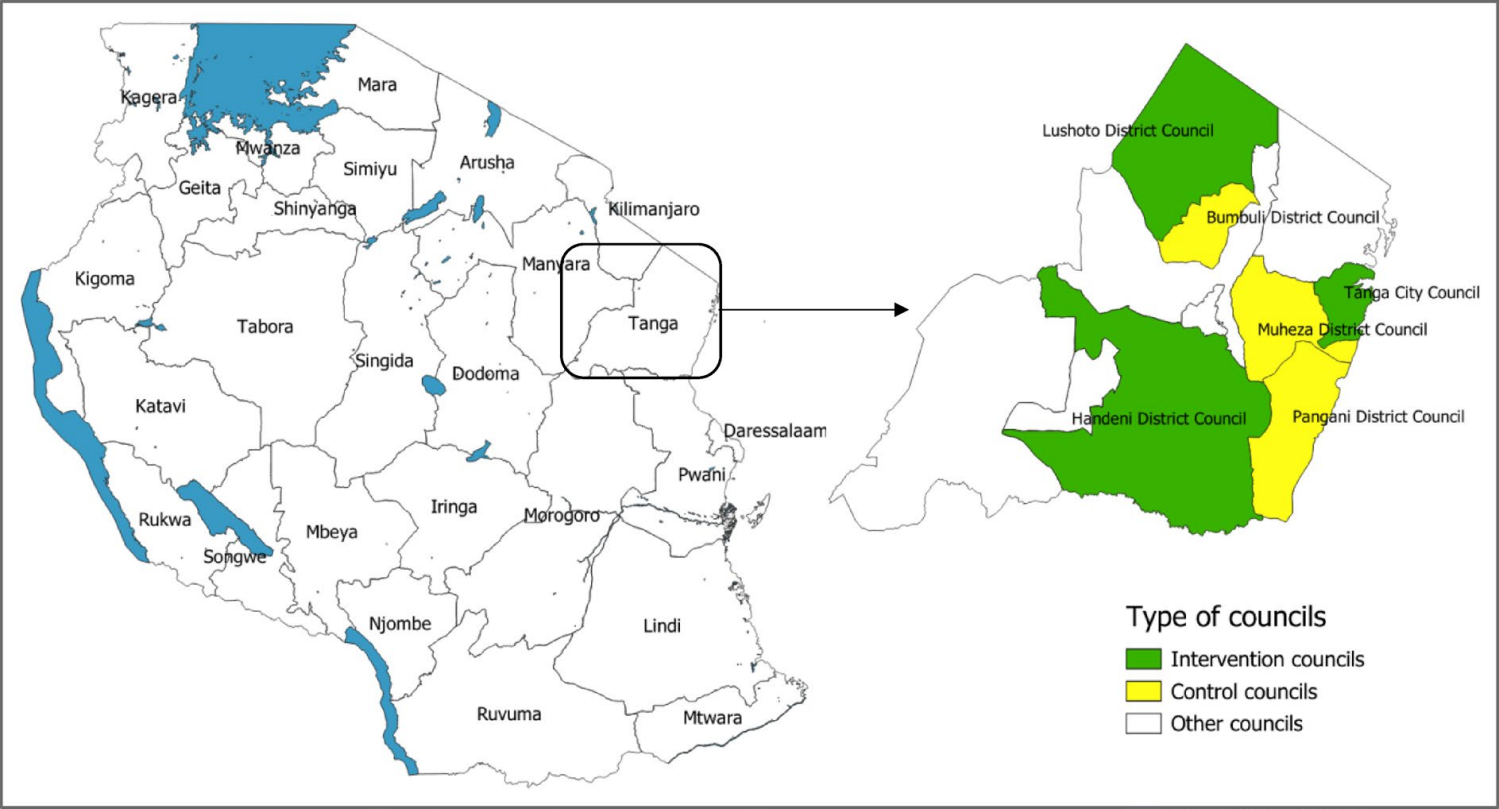
Map of Tanzania indicating the location of Tanga Region, the three councils where larviciding intervention was implemented (green shaded) and three control councils (yellow shaded). The map was produced in QGIS Desktop (version 3.40.8) using shapefiles obtained from the National Bureau of Statistics, Tanzania (https://www.nbs.go.tz/statistics/topic/gis).

This study was conducted in three intervention councils in Tanga Region (Handeni District Council DC, Tanga CC and Lushoto DC) and three selected control councils: Muheza DC, Bumbuli DC and Pangani DC. As mentioned earlier, the three intervention councils were selected pragmatically by the NMCP to represent different malaria risk strata (high, moderate and low malaria risk), as defined in the National Malaria Strategic Plan 2021–2025^43^. The control councils were selected based on their geographical similarity to intervention councils, in the same malaria risk strata. Muheza DC was paired with Handeni DC, Pangani DC was paired with Tanga CC, while Bumbuli DC was paired with Lushoto DC. The selected control councils were adjacent to the intervention councils, under the assumption that adjacent councils would share similar key characteristics, including vectorial systems. The selection of only three councils as controls was driven by cost considerations. The location of the Tanga Region and the six study councils in presented in Fig. 1.

### The larviciding intervention

The larviciding intervention in the three councils covered 91 villages in Handeni DC, 89 villages in Lushoto DC and 181 streets of Tanga CC. The population sizes of the councils were 384,353 for Handeni DC, 393,429 for Tanga CC, and 350,958 for Lushoto DC [21], giving a total population of 1,128,740 in the intervention area. The combined area of the three councils was 10,801 km², with individual sizes of 6,662 km² for Handeni DC, 3,534 km² for Tanga CC, and 605 km² for Lushoto DC^50^. Details regarding the number of CORPs engaged, and the breeding habitats identified and treated are reported elsewhere (Gavana et al.., submitted).

### Project implementation structure and institutional support

The larviciding intervention in Tanga Region was implemented entirely by the government system and using Community’s Own Resource Person (CORP) for mapping and larviciding. The programme was technically supported and supervised by the NMCP, in collaboration with the President’s Office - Regional Administration and Local Government (PO-RALG). Support was provided by the Towards Elimination of Malaria in Tanzania (TEMT) project implemented by the Swiss Tropical and Public Health Institute (Swiss TPH) and funded by the Swiss Government. The larviciding activities were conducted from April 2022 to March 2024.

### Biolarvicide products used

The project used two biolarvicide products, namely Bactivec^®^ with the spores of *Bacillus thuringiensis* var. *israelensis* (*Bti*) and Griselesf^®^ with the spores of *Bacillus sphaericus* (*Bs*). Both products are produced in-country by the Tanzania Biotech Products Limited (TBPL) (www.tanzaniabiotech.co.tz/*).* These bacteria-based larvicides produce mosquitocidal toxins that kill mosquito larvae at very low doses, while remaining completely safe to non-target organisms and the environment^51–54^. A study conducted in 2019 in both laboratory and semi-field settings by the National Institute for Medical Research (NIMR) confirmed that Bactivec^®^ and Griselesf^®^ applied at the recommended dose were efficacious against multiple species of mosquitoes up to seven days post-application^55^.

### Larviciding modality and frequency

The application of larvicides was implemented in rounds, based on local rainfall patterns. The three intervention councils experienced a bimodal rainfall pattern, with a heavy rainy season occurring in April and May and shallow rainy season in November and December. Dry season months were from June to October. Based on this pattern, three rounds of larvicide application were conducted per year: two months before the first heavy rainfall season, two months after the first heavy rainfall season, and two months before a shallow rainfall season. In one year, three larvicide application rounds of eight weeks each were conducted accounting for 24/52 or 46% of the weeks of a calendar year. This season-based, intermittent larviciding approach was adopted to save on the intervention costs compared to continuous application. With this modality, larvicide application was deliberately paused during periods of heavy rain to avoid product loss due to anticipated flushing by rainwater^13,56^.

The larvicides were rotated to mitigate against any potential development of resistance^13,57^. During each eight-week round, Bactivec^®^ (Bti) was applied in the first six weeks followed by Griselesf^®^ (Bs) in the last two weeks as Bs has a longer residual effect than Bactivec^®^ (Bti)^56,58^. Over the entire intervention period, six rounds of treatment were conducted, and a total of 64,000 L of larvicide were procured and applied.

### Entomological monitoring sites within councils

For this study, 20 monitoring villages were selected per council. The study councils varied in total area, population size, the number of wards and health centers (HCs), as shown in Supplementary Table 1. The villages or streets for entomological monitoring were selected based on the catchment areas of the health centers. This approach was intended to facilitate linkage between the entomological findings and epidemiological data obtained from health facilities, reported separately (Kailembo et al., submitted).

**Fig. 2:**
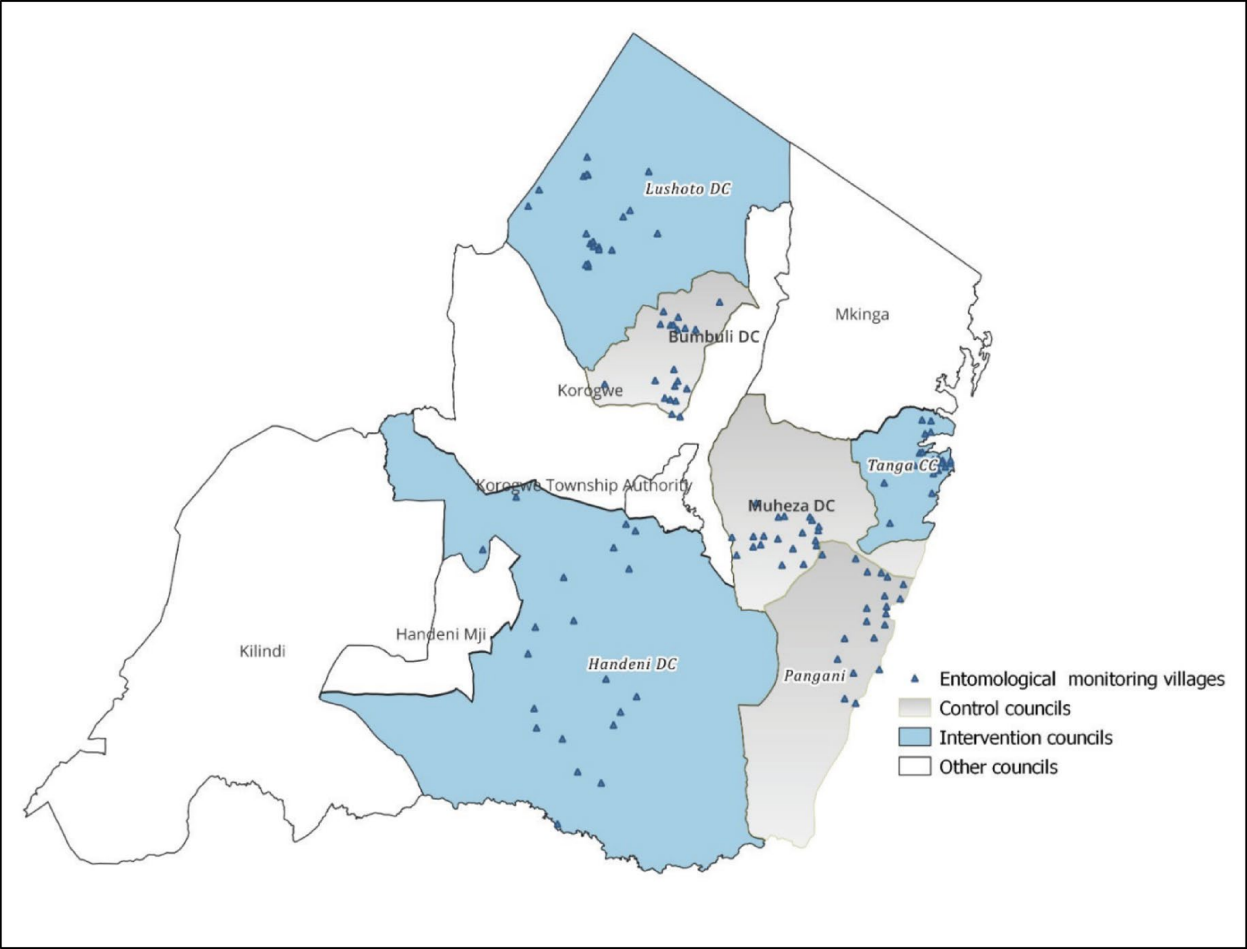
Map of Tanga Region indicating intervention and control councils including the location of sites (villages/streets) where the entomological monitoring was conducted. DC, District Council. CC, City Council. The map was produced in QGIS Desktop (version 3.40.8) using shapefiles obtained from the National Bureau of Statistics, Tanzania (https://www.nbs.go.tz/statistics/topic/gis). Global Positioning System (GPS) coordinates of study sites, collected by the study team, were incorporated into the map.

In all the study councils except Tanga CC, two HCs were purposively selected considering geographical representation and the distribution of their catchment villages. From each of the two selected HC catchment areas, 10 villages were purposively chosen based on health facility reports of malaria incidence and consultative guidance by the council and ward level health officials. For Tanga CC, streets were selected instead of villages. In urban areas of Tanzania, a “street” is a similar administrative entity to a village. Due to the clustering of health centers and the relatively small size of the council compared to the other five councils as shown in Supplementary Table 1, the selection of streets in Tanga CC was based on wards rather than health centers. Tanga CC comprises 27 wards in total. To select 20 streets, we first randomly selected 20 wards. Then, from each of these randomly selected wards, one street was purposively selected, based on consultative guidance from council and ward-level health officials. In total, the entomological monitoring was conducted in 120 sites across the six councils: 60 sites within intervention councils and 60 sites in control councils. The geographical distribution of selected sites across the study councils is shown in Fig. 2.

### Data collection

The entomological monitoring within intervention councils commenced in July 2022, while monitoring within the control councils followed one year later, in July 2023. This discrepancy was due to budget and logistical constraints. Entomological monitoring was conducted by trained and experienced technicians from the Ifakara Health Institute (IHI), each stationed in one of the six councils. Larvae and adult mosquito collections were carried out weekly and continuously, including during weeks without larvicide application, across both intervention and control councils. In each of the six councils, the collection of larval data was done in all 20 monitoring villages or streets, whereas the collection of adult mosquito data was only done in 16 villages selected randomly out of the 20, due to a limitation in the availability of mosquito traps.

### Collection of larvae

In each of the 20 monitoring villages, field officers assessed six breeding habitats per week. Hence, each council had a total of 120 breeding habitats assessed weekly, amounting to 720 breeding habitats across all six councils (the three intervention councils and three controls). The six breeding habitats in each village/street included four permanently monitored sites and two randomly selected sites. The selection of the four permanently monitored breeding habitats was based on the following criteria: (i) the habitat’s persistence throughout the dry season, and (ii) the presence of *Anopheles* larvae at the start of the follow-up. The random selection of the two additional habitats every week was done to account for any potential bias among the spraying personnel in taking extra efforts to apply biolarvicide in the assessed breeding habitats.

A breeding habitat was defined as standing water (ground, container or any object) able to support the growth of aquatic mosquito stages^59^. Breeding habitats could be as small as an animal footprint or as big as covering several square meters^60^. For this study, a breeding habitat was counted if it had an area of at least two square meters (m^2^) and was isolated from another water body by at least one meter. The habitats were categorized as open or closed. Open habitats included: puddles, tire tracks, swampy areas, mangrove swamps, drains, ditches, construction pits, foundations, man-made holes, water storage containers, seepages, springs, rice farms, other agricultural sources, streams and riverbeds, ponds, and pools. Closed habitats included: pit latrines, septic tanks, and soakage pits.

On every visit for larval assessment, information on the habitat’s type, geo-code, size, vegetation cover, larval occupancy and abundance was taken. Larval collection was conducted using standard dippers. The number of dips per breeding habitat was determined based on the surface area of the habitat. According to the WHO’s operational manual for larval source management, a footprint or hoofprint-sized habitat is considered equivalent to one dip. For larger breeding sites, such as borrow pits, it is recommended that one dip be taken per square meter^13^. In this study, we considered 5 dips for habitats with surface areas of 1–10 m², 10 dips for 11–100 m², and 15 dips for habitats larger than 100 m². The collected larvae were identified as belonging to either the anopheline sub-family or the culicine sub-family. The larvae were further classified based on their growth stage as being in the early stage (larval instar 1 and 2) or late stage (larval instar 3 and 4)^56^. The data were recorded using Open Data Kit (ODK) software -based forms on android tablets.

### Collection of adult mosquitoes

The Collection of adult mosquitoes was conducted at one sentinel house per village/street. In each of the 16 selected villages/streets per council, one sentinel house was selected for mosquito trapping. Selection of the houses was done upon consultative guidance by the village officials, with a focus on areas with the highest number of breeding habitats. In addition, preference was given to houses with structural features that facilitate mosquito entry, such as open eaves, mud walls, and unscreened windows^61–65^. Mosquito trapping was done only indoors using the Center for Disease Control Light Traps (CDC-LT). In each sentinel house, a Community’s Own Resource Person (CORP) set up the CDC-LT at 6 pm and collected the traps again the next morning at 6 am. The CORPs were instructed by the research team on how to set the CDC-LT, retrieve mosquitoes from the traps and preserve them. They were provided with traps and mosquito storage containers, prefilled with silica gel. The field officers collected the adult mosquitoes from the CORPs weekly, morphologically sorted the mosquitoes, counted them and entered the data into the ODK software.

### Morphological identification

Mosquitoes were morphological identified based on the criteria of Coetzee^66^. Male mosquitoes were counted, recorded and discarded. Female mosquitoes were further classified based on their abdominal status (unfed, partly fed, fully fed, and gravid). Female *Anopheles* mosquitoes were stored in Eppendorf tubes (1.5 ml) with silica gel desiccant and cotton wool and transferred to the IHI entomological laboratory in Bagamoyo for molecular analysis using Polymerase Chain Reaction (PCR) for speciation and detection of infection with *Plasmodium* sporozoites. All the female culicines were temporarily stored in pools for verification by supervisors before they were discarded.

### Laboratory analyses for species identification

Deoxyribonucleic Acid (DNA) was extracted from a wings or legs of each mosquito selected for analysis and amplified using multiplex PCR. The PCR assay was run in a 25 µl reaction volume containing 12.5 µl of 2x One Taq Quickload Master Mix (NEB M0486), 1 µl of each universal primer and specific reverse primers (10µM), 2 µl of extracted DNA and nuclease free water. For the *Anopheles gambiae* complex, the PCR targeted the genes at the Internal Transcribed Spacer region 2 (ITS 2) of the ribosomal DNA^67,68^. For *Anopheles funestus* sensu lato, the same gene (ITS 2) was targeted. The PCR products from *An.gambiae* and *An.funestus* species were visualized under UV light following electrophoresis on a 2% agarose gel stained with SafeView Classic. The mosquitoes that could not amplify for either *An. gambiae* species or *An. funestus* were subjected to *Anopheles stephensi*-specific primers for the detection of *An. stephensi* mosquitoes by real time quantitative qPCR assay. A total of 5,043 mosquitoes (62% of all anophelines collected) were tested for species identification.

### Sporozoite detection

DNA extracted from head and thorax of each mosquito was subjected to amplification for *Plasmodium* detection. Real-time quantitative PCR (qPCR) was performed using the Bio-Rad CFX Opus 96 system, with data analysis carried out using CFX Manager Software (version 3.1, Bio-Rad). Reactions were prepared using Luna^®^ Universal Probe 2X qPCR Master Mix (New England Biolabs, NEB M3004L). The multiplex qPCR assay simultaneously targeted two *Plasmodium*-specific gene sequences: the pan-*Plasmodium* 18 S rRNA gene (Pspp18S) and the *Plasmodium falciparum*-specific var gene acidic terminal sequence (*Pf*varATS). *Anopheles*-specific primer-probe set was included as an internal control to assess the quality of DNA extraction and amplification efficiency. Thermal cycling conditions were as follows: initial polymerase activation at 95 °C for 1 min, followed by 45 cycles of denaturation at 95 °C for 15 s, and annealing/extension at 57 °C for 45 s. Sporozoite detection was performed on all the 5,043 mosquitoes that were tested for species identification.

### Definitions of outcome variables

i. Larval density.

The total number of larvae per habitat and per dip. It was based on the total number of larvae collected, categorized as anophelines or culicines, their stages, as well as the number of dips taken per breeding habitat.

ii. Adult mosquito abundance.

The total number of adult mosquitoes collected per trap per area over a specified period. The adult mosquitoes collected weekly from the CDC-LT were sub-categorized into different mosquito species, i.e. *Anopheles gambiae* s.l., *Anopheles funestus* s.l., *Culex* species, *Aedes* species and other anophelines.

iii. Human biting rate (HBR).

The average number of bites from mosquitoes that a human receives over a specific period, often measured per person per night. The collection of adult mosquitoes in this study was conducted using CDC-LT.

iv. Sporozoite rate (SR).

The percentage of mosquitoes that are infected with sporozoites, the infectious stage of the malaria parasite.

v. Entomological inoculation rate (EIR).

The number of infectious bites a person receives from mosquitoes over a given period.

### Data analyses

The different types of breeding habitats assessed were summarized using proportions. Trends in the density of larvae, and in the abundance of adult mosquitoes by different species and per strata were plotted. The HBR was calculated by dividing the total number of female mosquitoes collected, by the product of the total number of traps and the number of trapping nights. Here, “number of traps” refers to the total count of CDC-LTs deployed per night across different sites, while trapping nights denote the cumulative number of nights on which mosquito collections were conducted across all sites. CDC-LTs are widely accepted as a safer and more practical alternative to Human Landing Catch (HLC) for measuring indoor malaria transmission risk in Africa^69^. In this study, the number of mosquitoes captured using CDC-LTs was considered equivalent to the number that would be caught via HLC. This approach for estimating HBR using CDC-LTs has been applied in previous studies^70,71^.

The SR was calculated by dividing the number of mosquitoes found to have *Plasmodium* sporozoites by the total number of mosquitoes that were tested^69,70^. The EIR was calculated by multiplying the HBR and the SR.

In order to estimate the association between the intervention and the density of either larvae or mosquito abundance, we used a negative binomial regression. Larvicide application was coded as a binary variable and counted as being applied only during the weeks of application. Council was included as a fixed effect. Additional covariates were included to adjust for potential confounding: duration / time of the study; ITN access; climate data including rainfall (in mm) and maximum temperature (in degree Celsius). Rainfall and temperature data were obtained from the Tanzania Meteorological Authority (TMA), while ITN access was derived from the literature^72^. Temperature, rainfall, and ITN access were treated as categorical variables as they did not exhibit a linear relationship with the outcome. An autoregressive term on mosquito counts with a two-week lag was included to account for the time series nature of the data and their temporal autocorrelation, i.e., that mosquito populations from the previous two weeks impact the current counts of mosquitoes. To capture non-linear temporal trends, we modeled time using a natural spline. The analyses were carried out in R using R Studio version 4.4.1.

## Results

### Types of breeding habitats

**Fig. 3:**
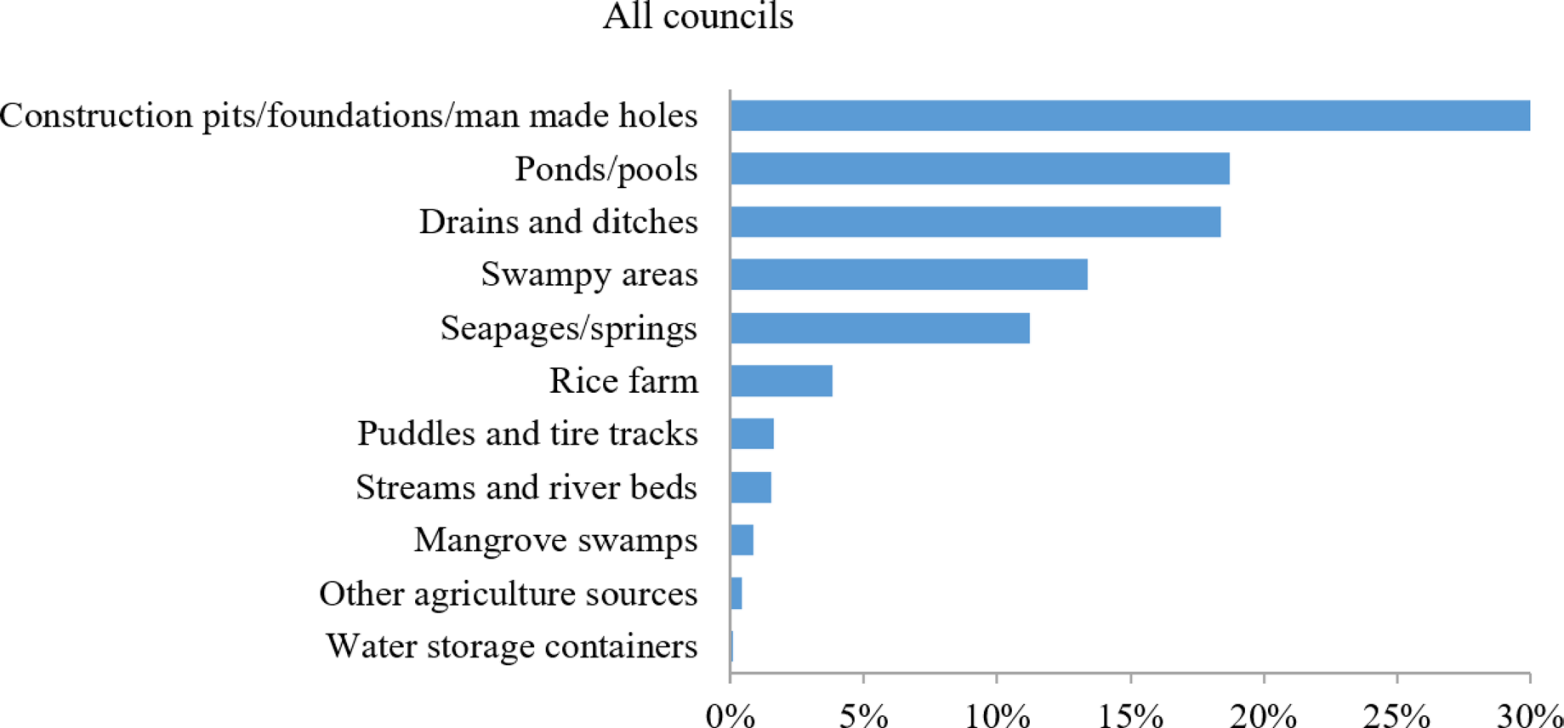
Types of surveyed breeding habitats across all councils.

Of all the breeding habitats visited, man-made holes, ponds/pools and drains and ditches were the top three most common habitat types (Fig. 3).

### The density of larvae

**Fig. 4:**
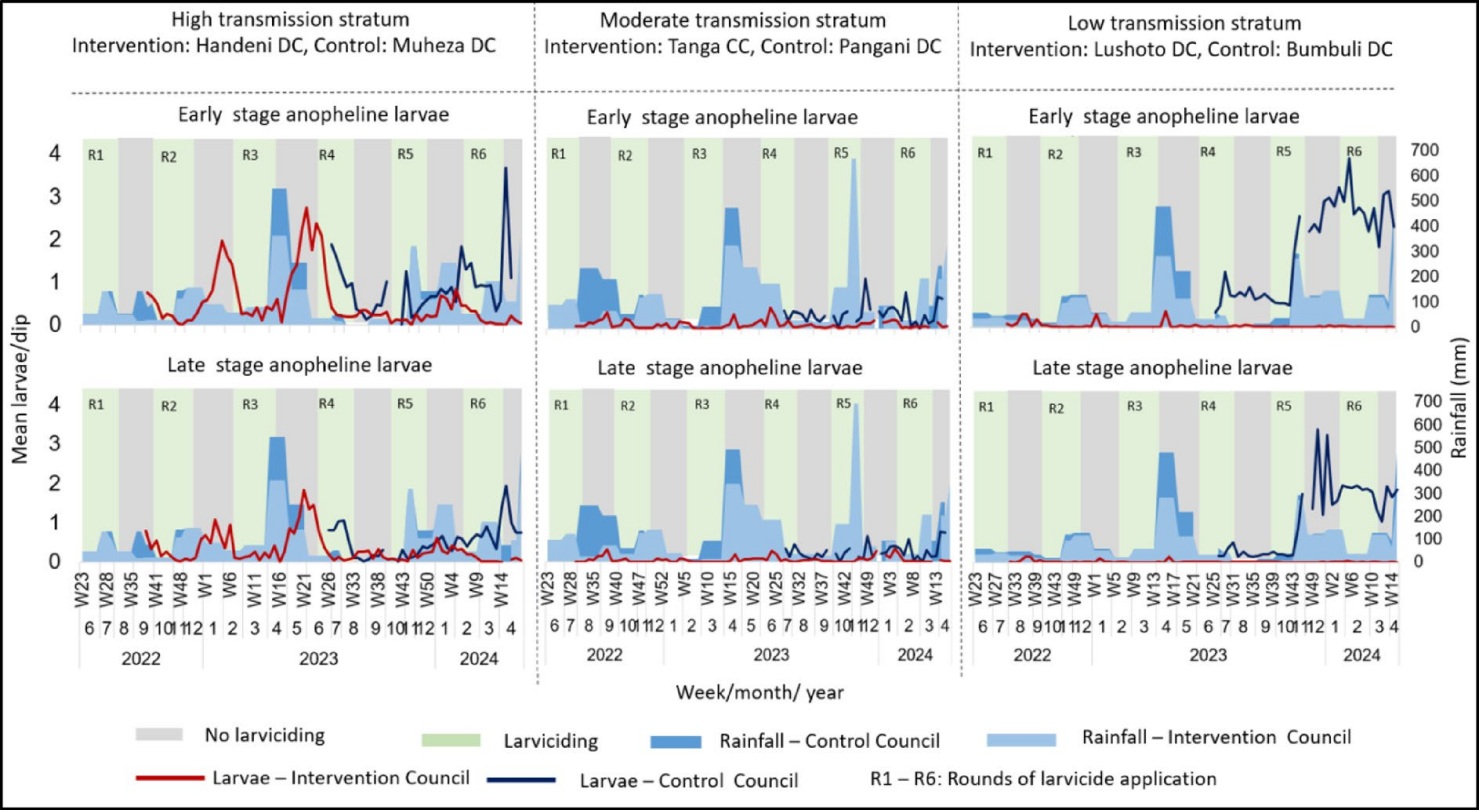
Anopheline larvae densities in the intervention and control councils, by malaria transmission strata. Larviciding rounds are indicated by light green columns and numbered R1 to R6. Grey columns indicate periods of no larviciding. Blue bars highlight rainfall data. The lines indicate weekly larvae densities, and the gaps on the trend lines indicate weeks with no data collection due to heavy rains or logistical challenges.

For anophelines (Fig. 4) larviciding was associated with reductions in larval densities across all transmission strata. In the high transmission stratum, early and late-stage larvae declined during larviciding rounds, but densities rebounded when larviciding was paused, reaching over 3 larvae/dip post-R6. In the moderate transmission stratum, larval densities in the intervention council remained low (< 0.5 larvae/dip) throughout, while control council densities rose steadily, peaking above 3 larvae/dip by early 2024. In the low transmission stratum, larval densities in the intervention council remained near zero, contrasting with sharp increases in the control councils, particularly during the rainy season. Across all strata, larval resurgence aligned with periods of heavy rainfall and gaps in larvicide application.

**Fig. 5:**
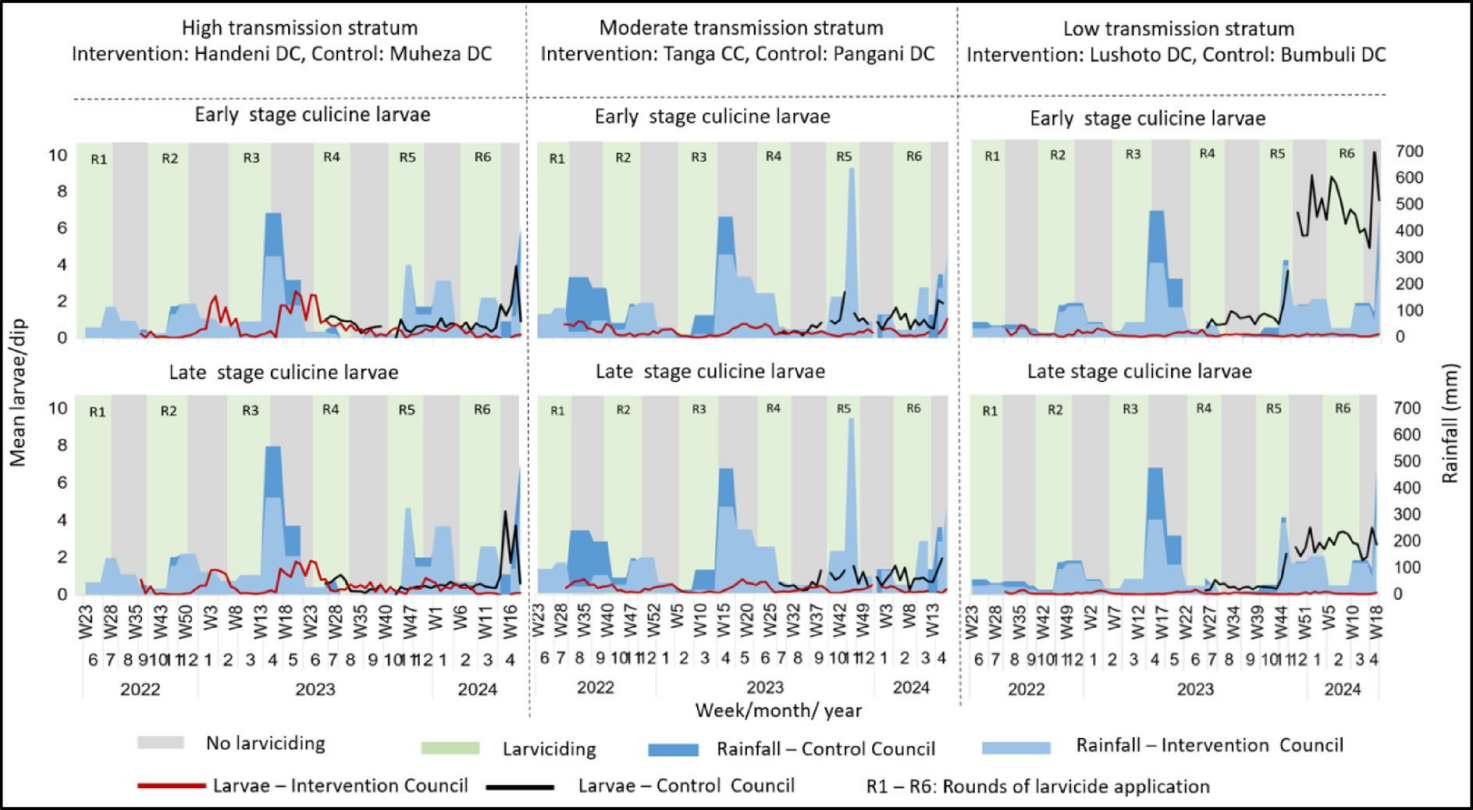
Densities of culicine larvae in the intervention and control councils, across the three malaria transmission strata. Larviciding rounds are indicated by light green columns and numbered R1 to R6. Grey columns indicate periods of no larviciding. Blue bars highlight rainfall data. The lines indicate weekly trends of larvae densities and the gaps on the trend lines indicate weeks with no data collection due to heavy rains or logistical challenges.

For culicines (Fig. 5), larviciding was also associated with marked reductions in larval densities across all transmission strata. In the high transmission stratum, the early and late-stage larvae declined during larviciding rounds in the intervention council and remained near zero for most of the monitoring period, while densities in the control council increased sharply during the rainy season, peaking above 8 larvae/dip by early 2024. In the moderate transmission stratum, larval densities in the intervention council stayed low (< 0.5 larvae/dip) throughout, whereas control council densities rose steadily during wet periods, reaching over 6 larvae/dip in late 2023 and early 2024. In the low transmission stratum, intervention council densities for both early- and late-stage larvae were consistently minimal, in contrast to the control council where large peaks occurred during the rainy season, exceeding 9 larvae/dip in early 2024.

### The abundance of adult mosquitoes

**Fig. 6:**
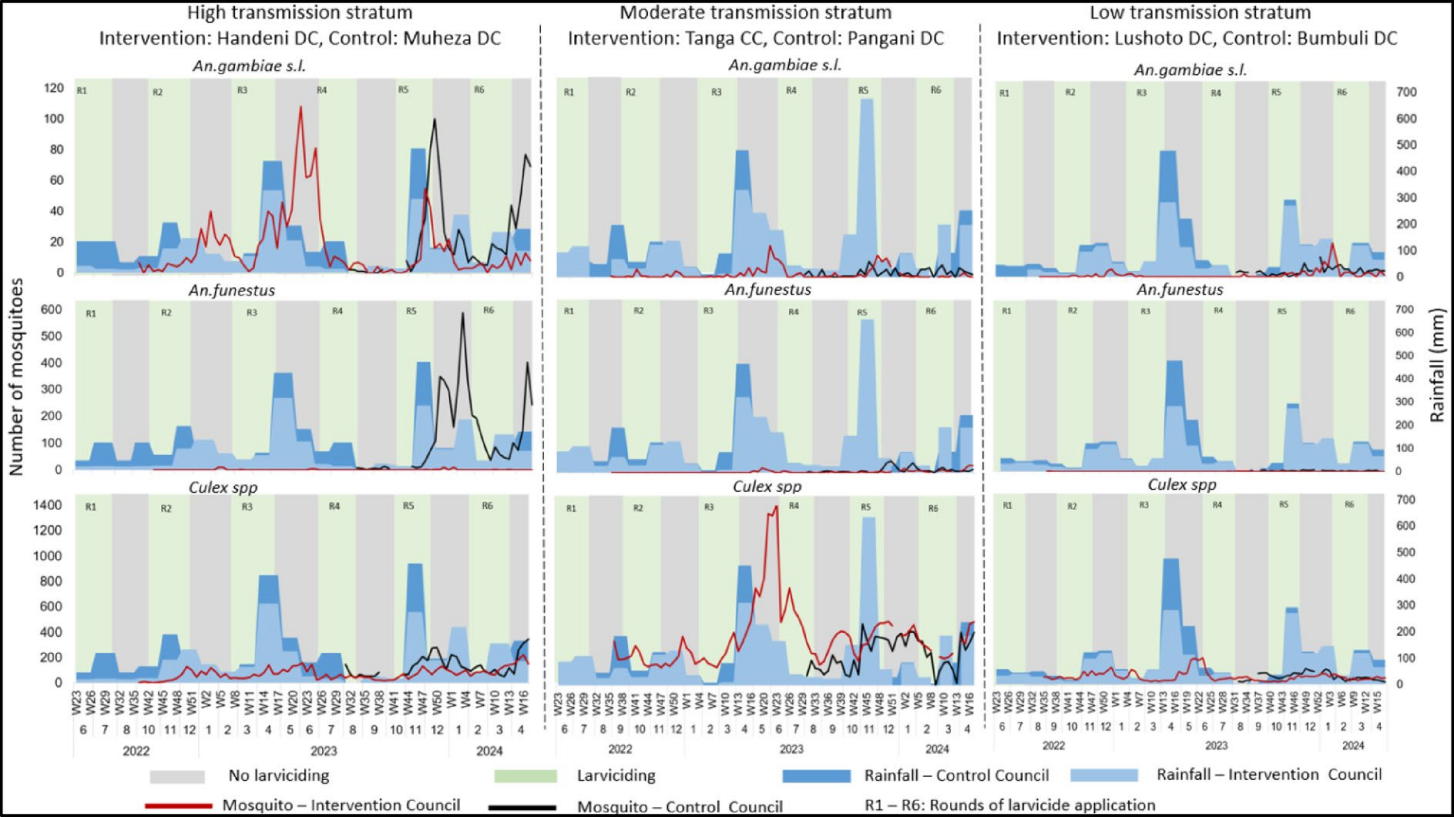
Abundance of adult mosquitoes per species in the intervention and control councils, across the three malaria transmission strata. Larviciding rounds are indicated by light green columns and numbered R1 to R6. Grey columns indicate periods of no larviciding. Blue bars highlight rainfall data. The lines indicate weekly trends of mosquito abundance, and the gaps indicate weeks with no data collection due to logistical challenges.

A total of 68, 676 mosquitoes including 67,372 (98%) females and 1,304 males were collected across the intervention and control areas. Among the 67,372 female mosquitoes, 8,106 (12%) were anophelines and 59,266 (88%) were culicines. Similar to the trends seen in the larvae, the data for adult mosquitoes suggested that there was a lower number of mosquitoes during the period of larvicide application compared to the period without larviciding (Fig. 6). However, the trends were difficult to appreciate in the low-risk strata due to the low numbers of adult mosquitoes collected.

### Association between larviciding and larval densities

The estimates for the association between larviciding and anopheline larvae by means of a negative binomial regression were generally in the direction of a reduction. For early-stage anopheline larvae, a significant decrease in the larval abundance was observed only in the high-risk stratum while for the late-stage anopheline larvae a significant reduction was found across all three malaria risk strata (Table 1). For the culicine larvae, most estimates were in the direction of lower abundance during larviciding, however, they were significant only for late-stage larvae in the high and moderate-risk strata (Table 1).

**Table 1:** Estimated associations between larviciding and larvae abundance (negative binomial regression). Note: DC = District Council; CC = City Council; IRR = Incidence rate Ratio.

Larvae species	Larval stage	High risk Handeni DC (Intervention) vs. Muheza DC (Control)		Moderate risk Tanga CC (Intervention) vs. Pangani DC (Control)		Low risk Lushoto DC (Intervention) vs. Bumbuli DC (Control)	
		IRR (95% CI)	p-value	IRR (95% CI)	p-value	IRR (95% CI)	p-value
Anopheline	Early	0.38 (0.22; 0.64)	< 0.001	0.94 (0.46; 1.89)	0.845	0.78 (0.50; 1.21)	0.277
	Late	0.20 (0.12; 0.35)	< 0.001	0.51 (0.26; 1.01)	0.041	0.40 (0.24; 0.68)	< 0.001
Culicine	Early	0.70 (0.40; 1.22)	0.194	0.61 (0.34; 1.09)	0.085	1.14 (0.83; 1.58)	0.433
	Late	0.49 (0.28; 0.84)	0.008	0.31 0.17; 0.54	< 0.001	0.86 (0.61; 1.22)	0.392

### Association between larviciding and the abundance of adult mosquitoes

There was a significantly lower abundance for *An. gambiae* s.l. and *Culex* spp. in the moderate and low malaria risk strata, as determined by negative binomial regression. However, there was no evidence of lower abundance in any of the mosquito groups in the high transmission stratum. There was also no evidence of any associations in *An. funestus* across any of the strata (Table 2).

**Table 2:** Estimated associations between larviciding and abundance of adult mosquitoes (negative binomial regression). Note: DC = District Council; CC = City Council; IRR = Incidence rate Ratio.

Mosquito species	High risk Handeni DC (Intervention) vs. Muheza DC (Control)		Moderate risk Tanga CC (Intervention) vs. Pangani DC (Control)		Low risk Lushoto DC (Intervention) vs. Bumbuli DC (Control)	
	IRR (95% CI)	*p*-value	IRR (95% CI)	*p*-value	IRR (95% CI)	*p*-value
*An. gambiae* s.l	0.94 (0.37; 2.43)	0.901	0.09 (0.02; 0.32)	< 0.001	0.29 (0.10; 0.78)	0.020
*An. funestus s*.l	1.96 (0.29; 11.20)	0.439	1.05 (0.32; 3.47)	0.932	8.82 (0.81; 95.77)	0.074
*Culex* spp	1.343 0.762; 2.377	0.307	0.80 (0.76; 0.85)	< 0.001	0.54 (0.34; 0.86)	0.010

### Mosquito species composition

Of the 5,043 female anophelines that were subjected to PCR for species identification 5,005 (99%) amplified while 38 mosquitoes did not amplify. All 38 mosquitoes that did not amplify with primers for *An.gambiae* s.l. or *An. funestus* s.l were subjected to *An. stephensi* primers, but all tested negative. Of the 5,005 mosquitoes that amplified, *An. funestus* s.l constituted the largest proportion (60.0%). Within the *An. funestus* complex, *An. funestus sensu stricto* was the dominant sibling species (89.1%). In the *An. gambiae* s.l complex, *Anopheles arabiensis* contributed the largest proportion (51.9%) of the sibling species (Supplementary Table 2). The composition of the mosquito species varied across the malaria risk strata and councils within the strata (Supplementary Table 3).

### Sporozoite rate (SR) and entomological inoculation rate (EIR)

The SR and EIR differed among strata and councils. Each council’s values are shown for all *Anopheles* species combined and then broken down by the two major malaria vector groups: *An. gambiae* s.l. and *An. funestus* s.l. in Supplementary Table 4.

## Discussion

This study assessed the association between a large-scale larviciding intervention and key entomological indicators by comparing three pilot councils representing different malaria risk strata, with three control councils in the Tanga Region. Most of the mosquito breeding habitats identified during the study were man-made, including construction pits/foundations/man-made holes/wells, ditches and trenches. This observation is consistent with previous reports^5,73,74^, which reported that water bodies resulting from human activities accounted for a large proportion of mosquito breeding habitats. However, a large part of the study area was located in rural settings, where a higher proportion of natural water bodies would be expected. This pattern might have reflected the criteria used to select habitats for monitoring. Four out of the six monitored breeding sites were selected based on their semi-permanent to permanent nature. As a result, many natural but temporary habitats may have been excluded from the sample.

Nevertheless, the presence of productive, man-made permanent habitats highlighted an important role of human activity in sustaining mosquito breeding. In the context of an intermittently implemented larviciding, these permanent habitats are particularly concerning as they may continue to support mosquito populations during periods when larvicide application is paused. On the positive side, the individuals or communities responsible for their creation can potentially be engaged in their control. This further reinforces the WHO’s recommendation that LSM should be implemented through community-based approaches, as habitats created by human activities can often be effectively managed by the individuals or communities responsible for their creation^13^.

Using community members to identify and eliminate sources of mosquitoes can be cheaper, more effective and sustainable than when implemented by the government^59,75–79^. However, the Tanga project focused primarily on larviciding, with limited emphasis on other LSM strategies, such as habitat modification and habitat manipulation. The key operational components of larviciding, particularly the identification of breeding habitats and the application of larvicides on a weekly basis, were conducted by two CORPs per village. Relying on only two individuals per village for weekly larviciding, especially in areas where breeding sites are numerous, dynamic, and difficult to locate is not optimal because this work is labor-intensive and exceeds what volunteers can be expected to do in the long-term (Kihwele et al.. submitted). Unfortunately, increasing the number of CORPs will substantially increase the overall implementation cost. To address this limitation, the integration of advanced technologies such as drones and geospatial mapping tools could potentially improve habitat detection, particularly in hard-to-reach areas and during peak breeding seasons^22,30,80^. However, these technologies have never been tested in areas as large as the one reported here, and the technical capacities required to make the best use of these technologies are above the current capacity at council and national level.

In such settings, incorporating environmental management strategies such as environmental modification and manipulation in the frame of a multisectoral approach could reduce the number of breeding habitats, hence requiring later less larvicide application, thereby enhancing operational efficiency and sustainability.

This study found that the larviciding intervention in Tanga Region was associated with a decline in both larval and adult mosquito densities during the weeks of larvicide application in some species and risk strata. There were significantly lower densities of late-stage larvae of all the mosquito species across all the strata, except culicine species in the low malaria risk stratum. For adult mosquitoes there were lower *An. gambiae* s.l. and *Culex* species densities associated with the larviciding intervention in the low and moderate risk strata, but not in the high-risk stratum.

Similar to the findings of the current study, previous studies conducted in different settings across Africa have reported variable outcomes regarding the impact of larviciding interventions on the reduction of larval and adult mosquito populations. While some studies have demonstrated that larviciding significantly reduced adult mosquito densities^56,81–85^, others have reported variable effects depending on mosquito species and ecological or operational contexts^14,47,86,87^. These findings, which show the variability in the effectiveness of larviciding across African settings, underscore the importance of adopting context-specific strategies when implementing larviciding^18,19,88^.

The findings of this study suggest that larviciding could serve as a potentially valuable complementary intervention for mosquito control, particularly if intervention strategies are optimized. However, in the case of the Tanga Region, although the intervention led to significant reductions in larval and adult mosquito populations, these gains were short-lived. A rapid rebound across all mosquito stages was observed shortly after the larvicide application was halted. This rebound was especially pronounced among adult mosquitoes of both *Anopheles* and culicine species between rounds 3 and 4 (during the months of April and May), a period characterized with heavy rainfall, during which larvicide application was deliberately paused to avoid the anticipated flushing out of the product^56,58^. Such rainfall likely created optimal conditions for mosquito proliferation by enabling the formation and persistence of standing water suitable for breeding^89–91^. This rapid resurgence of mosquito populations during untreated periods underscores the need to move to continuous larviciding, guided by short-term rainfall forecasts, to enable timely responses to mosquito breeding surges and enhance the overall effectiveness of the intervention.

In future, the use of longer-lasting larviciding approaches could offer operational advantages by reducing the frequency of application and maintaining suppression over extended periods. For example, formulations based on insect growth regulators (IGRs) such as pyriproxyfen^92–95^, or slow-release formulations of Bti^96–98^ have demonstrated extended residual activity ranging from several weeks to months, depending on environmental conditions. These products may be particularly useful in settings where frequent reapplication is logistically or financially challenging. However, longer-lasting formulations may come with trade-offs, including higher upfront costs, potentially lower immediate larvicidal efficacy, and limited field evidence in an ecological context like Tanga.

Another important risk is the likelihood of missed breeding habitats during the initial mapping. Missed sites will obviously remain untreated, thereby sustaining mosquito populations. Achieving a high coverage in the identification and treatment of mosquito breeding habitats is a critical factor in the effectiveness of larviciding interventions^13,99^. Although we could not independently determine the percentage of all breeding sites that were found and treated (and thus also the percentage that were missed), a cross-sectional study (Gavana et al. unpublished) showed that CORPs were able to detect only 51% of all habitats compared to those identified by the research team. Reasons for the CORPs failure to identify all habitats in this study could be related to the large size of the intervention areas, and a difficult access to some locations as reported by Kihwele et al. (submitted). From the study on LSM conducted in Dar es Salaam 20 years ago, CORPs detected only 41% of breeding habitats during the baseline^100^ and 66% during the implementation phase^45^. The main reasons for missing breeding habitats in Dar es Salaam included unfamiliarity of the CORPs with the areas and inaccessibility of locations, especially those within fenced properties^45^.

Deploying a team of experts to verify the sites reported by CORPs could help minimize the risk of missed habitats, thereby increasing the coverage and presumably enhancing the overall impact of the intervention. Additionally, periodic refresher training and supportive supervision aimed at improving habitat identification coverage are essential. The importance of robust vertical oversight and monitoring of LSM activities is emphasized^101^.

In this study, significant reductions were found in the populations of adult *An. gambiae* s.l and *Culex* species within the moderate and low strata. However, no reductions were found in the high malaria risk stratum. This may be due to the high number of breeding habitats, since the Handeni Council is a rural district with a large geographical area (Kailembo et al., submitted). With such large geographical areas and a high number of breeding habitats, the logistical burden of the intervention is considerable.

Another finding was that there was the absence of an association between the larviciding intervention and the densities of *An. funestus* across all of the malaria risk strata. This may be attributed both to the ecology of its breeding habitats, and the intermittent larviciding modality of this project. Previous studies conducted in southeastern Tanzania described *An. funestus* breeding habitats as permanent to semi-permanent water bodies with clear water and vegetation present year-round^87,102,103^. Importantly, the breeding habitats covered with vegetation are operationally difficult to reach or effectively treat with a liquid biolarvicide^88,104^. In such areas where breeding habitats are covered with vegetation and larvicide application relies on hand-operated knapsack pumps, the use of motorized pumps or granular larvicide formulations should be recommended^86^. However, in the Tanga project, larvicide application relied entirely on hand-operated knapsack pumps, raising the possibility that habitats colonized by *An. funestus* were ineffectively treated. Besides being highly resistant to pyrethroids^105–111^, *An. funestus* is recognized as an efficient malaria vector, responsible for a substantial proportion of ongoing malaria transmission in Tanzania^105,112–115^.

The results show a clear variability in the distribution and species composition of mosquitoes. Within the *An. gambiae* complex, *An. arabiensis* was dominant in Lushoto DC while *An. gambiae* s.s. prevailed in Muheza and Pangani DCs. Handeni DC exhibited a unique mix of *An. arabiensis* and *An. merus*. The high proportion of *An. arabiensis*, a predominantly outdoor-biting species^13^, supports vector control strategies that extend beyond indoor interventions. Meanwhile, the presence of *An. merus*, which breeds in brackish water^116^ in Handeni DC poses operational questions about the effectiveness of larviciding in such settings, warranting further investigation. The presence of multiple important vector species with differing ecological and behavioral traits in Handeni DC may be the reason for persistently high malaria transmissions in the area, underscoring the need for ecologically informed vector control approaches.

The strength of this study is the independence nature of the evaluation team, composed of trained scientists operating independently from the intervention implementers. The use of control councils provided a basis for comparison of trends over time, although these were only monitored in the second year of the intervention. Furthermore, the longitudinal monitoring of both larvae and adult mosquitoes across periods with and without larvicide application enabled the capture of weekly mosquito density trends under varying intervention conditions, documenting for example the re-bound of larvae in the in-between larviciding weeks.

However, the interpretation of the current study results should take into account some limitations. Only one intervention and one control council were selected per stratum, which limited the ability to assess intra-stratum variability. Also, the selection of intervention councils was pragmatic rather than randomized.

Visual inspection of the descriptive results of adult mosquito densities indicated that levels of *Culex* spp. in both the intervention and control areas were comparable in the moderate transmission settings. This was an unexpected finding, which requires careful examination. On one hand, it is important to note here again that the intervention and control areas within the moderate transmission setting differed not only in terms of intervention status but also in some demographic and ecological characteristics that could explain the observed pattern of *Culex* spp. densities. On the other hand, the main aim of the larviciding operation was to control malaria vectors and some typical peri-domestic *Culex* spp. breeding sites with polluted water such as pit latrines could have been overlooked.

Multivariable analysis revealed that larviciding was associated with a statistically significant reduction of *Culex* spp, with an Incidence Rate Ratio (IRR) of 0.80 (95% CI: 0.76–0.85; *p* < 0.001). This was after adjusting for potential confounding variables such as temperature, rainfall, and ITN coverage. The apparent discrepancy between the visual results and the statistical findings highlights the importance of a multivariate analysis, controlling for underlying covariates that may mask the effect of the intervention when descriptive results are presented. The statistical results suggest that the larviciding intervention had a measurable impact on mosquito densities, despite not being apparent in the un-adjusted comparisons.

We noted that in the moderate transmission stratum, larviciding had a greater impact on larval-stage culicines than on adults. Our results from laboratory evaluations of the products proved that the larvicides were efficacious against multiple species of mosquito larvae, including *Culex* spp. The relative lack of impact on adult populations may be explained amonng other factors by the low coverage in identifying and treating breeding habitats that supported *Culex* proliferation. The moderate risk intervention council (Tanga City Council) is an urban area harbouring numerous and diverse *Culex* breeding habitats (such as poorly managed drainage systems, septic tanks, pit latrines, etc.) compared to rural settings . These habitats were more difficult to locate or access, and were not the primary target of our larviciding programme. This is likely to have contributed to the poor effects on adult *Culex* populations.

Another notable limitation of this study was the fact that the entomological monitoring in the control council was initiated one year after monitoring had already commenced in the larviciding councils. So, a direct comparison between intervention and control districts was only possible for the second year of intervention.

Given the importance of year-on-year fluctuations in key vector parameters, this diminished the reliability of the intervention-control comparison. Having had two years of entomological data in both the intervention and control councils would have benefitted our evaluation by giving a more robust comparative analysis. This would have allowed for stronger attribution of observed entomological changes to the intervention itself, rather than to environmental or seasonal variation. The latter were included as co-variates in our analysis, but that could almost certainly not neutralize completely their effect.

Adding a baseline data collection of one year would have also made our comparative analysis stronger, with a more robust difference-in-difference (DiD) analytical approach^117,118^ but unfortunately this was not materially possible. The large scale of the larviciding operation required a large-scale monitoring setup, and no resources for a baseline year were available.

Furthermore, the results showed that there was consistently low vector densities of *An. funestus* in the intervention areas throughout the study period, giving an impression that the intervention was effective in reducing the abundance of this vector. This is contrary to the findings from our multi-variable statistical analysis which showed that larviciding did not seem to have an effect on *An. funestus* densities. The control areas had substantially higher densities of *An. funestus* before the start of the intervention, and this difference remained throughout the study period. Thus, the observed difference is most likely attributable to pre-existing disparities between the intervention and control areas, rather than the effect of the intervention itself. Future evaluations of larviciding interventions should include the collection of baseline entomological data and particularly have more units to compare, to ensure a more valid comparison of intervention effects.

## Conclusions

The current study provides evidence that the larviciding intervention in Tanga Region contributed to reductions in late-stage mosquito larvae and adult *An. gambiae* s.l. and *Culex* populations in moderate and low-risk areas. However, the intervention was not associated with any reductions in *Anopheles* mosquitoes in the high-risk strata and generally there was no effect on *An. funestus* populations, which may limit its overall impact on malaria transmission. These findings suggest a potential role of larviciding as a complementary vector control tool in selected settings in Tanzania, but not everywhere. Continuous larviciding, increased community involvement, better coverage of breeding habitats, and deploying approaches tailored to local vector ecologies particularly for *An. funestus* and *An. merus* will be critical for optimizing the effectiveness of the larviciding intervention.

## Supplementary Information

Below is the link to the electronic supplementary material.

Supplementary Material 1

## Data Availability

Data can be made available upon receipt of official reasonable requests to the corresponding author and with approval from the TEMT project director and the Government of Tanzania.

## Abbreviations

Bti: *Bacillus thuringiensis* var* israelensis*
Bs: *Bacillus sphaericus*
CC: City council
CDC-LT: Center for disease control light trap
CORPs: Community owned resource persons
DC: District council
DNA: Deoxyribonucleic acid
EIR: Entomological inoculation rate
HC: Health Centre
IHI: Ifakara Health Institute
ITNs: Insecticide treated-mosquito nets
IRS: Indoor residual spraying
IRR: Incident rate ratio
ITS: Internal transcribed spacer
LSM: Larval source management
NIMR: National Institute for Medical Research
NMCP: National malaria control program
ODK: Open data kit
PCR: Polymerase chain reaction
PO-RALG: President’s Office – Regional Administration and Local Government
SR: Sporozoite rate
Swiss TPH: Swiss Tropical and Public Health Institute
TBPL: Tanzania biotech products limited
TEMT: Towards elimination of malaria in Tanzania
UMCP: Urban malaria control program
VEO: Village executive officer
WEO: Ward executive officer
WHO: World Health Organization
