# Supplementary material for: A large-scale mosquito larviciding in Tanga Region, Tanzania, reduced mosquito densities to varying degrees across malaria transmission risk strata
Source: Sci Rep. 2025 Nov 27;15:42461. doi: 10.1038/s41598-025-26675-w (PMC12660774; doi:10.1038/s41598-025-26675-w)
Supplement: Supplementary file 1 — Supplementary Material 1 [file 41598_2025_26675_MOESM1_ESM.docx]

**Supplementary information**

**Supplementary Table S1**. Characteristics of the councils and villages selected. Total land area (in km^2^) , the number of wards and HCs in the intervention and control councils, total population per councils according to the 2022 general population census [^52^](#_ENREF_52) and the number of villages/streets selected for the study. Note: DC = District Council; CC = City Council.

| Council name | Number of wards | Number of public health Centers | Total area in km^2^ | Total population | Villages /streets selected |
| --- | --- | --- | --- | --- | --- |
| **Intervention** | | | | | |
| Handeni DC | 21 | 3 | 6,662 | 384,353 | 20 |
| Lushoto DC | 33 | 5 | 3,534 | 350,958 | 20 |
| Tanga CC | 27 | 7 | 605 | 393,429 | 20 |
| **Total** | **81** | **15** | **10,801** | **1,128,740** | **60** |
| **Control** | | | | | |
| Muheza DC | 37 | 2 | 1,545 | 238,260 | 20 |
| Bumbuli DC | 18 | 4 | 805 | 159,373 | 20 |
| Pangani DC | 14 | 2 | 1,815 | 75,642 | 20 |
| **Total** | **69** | **8** | **4,165** | **473,275** | **60** |

**Supplementary Table S2**. Sibling mosquito species within complexes. Note. PCR = Polymerase Chain Reaction.

| **Complex** | **Sibling species identified using PCR** | **Number** | **% across all complexes** | **% in**  ***An. funestus* complex** | **% in**  ***An. gambiae* complex** |
| --- | --- | --- | --- | --- | --- |
| *An. funestus* s.l | *A. funestus* s.s | 2,669 | 53.3 | 89.1 | na |
|  | *An. parensis* | 21 | 0.4 | 0.7 | na |
|  | *An. rivulorum* | 305 | 6.1 | 10.2 | na |
| *An. gambiae* s.l | *An. arabiensis* | 1,043 | 20.8 | na | 51.9 |
|  | *An. gambiae* s.s | 463 | 9.3 | na | 23.0 |
|  | *An. merus* | 504 | 10.1 | na | 25.1 |
| Total |  | 5,005 | 100 | 100 | 100 |

**Supplementary Table S3**. Species composition, by malaria risk strata. Note: DC = District Council; CC = City Council

| **Species** | **High risk** | | **Moderate risk** | | **Low risk** | |
| --- | --- | --- | --- | --- | --- | --- |
|  | **Handeni DC** | **Muheza DC** | **Tanga CC** | **Pangani DC** | **Lushoto DC** | **Bumbuli DC** |
|  | **n (%)** | **n (%)** | **n (%)** | **n (%)** | **n (%)** | **n (%)** |
| ***An. funestus* s.l** | | | | | | |
| *An. funestus* s.s | 81  (92.0%) | 2,021  (88.0%) | 301  (90.7%) | 225  (96.2%) | 4  (57.1%) | 38  (97.4%) |
| *An. parensis* | 1  (1.1%) | 18  (0.8%) | 0  (0%) | 1  (0.4%) | 0  (0%) | 1  (2.6%) |
| *An. rivulorum* | 6  (6.8%) | 257  (11.2%) | 31  (9.3%) | 8  (3.4%) | 3  (42.9%) | 0  (0%) |
| Total | 88  (100%) | 2,296  (100%) | 332  (100%) | 234  (100%) | 7  (100%) | 39  (100%) |
| ***An. gambiae* s.l** | | | | | | |
| *An. arabiensis* | 542  (45.0%) | 198  (46.8%) | 78  (53.1%) | 2  (18.2%) | 220  (97.8%) | 1  (100%) |
| *An. gambiae* s.s | 178  (14.8%) | 212  (50.1% | 62  (42.2%) | 9  (81.8%) | 5  (2.2%) | 0  (0%) |
| *An. merus* | 484  (40.2%) | 13  (3.1%) | 7  (4.8%) | 0  (0%) | 0  (0%) | 0  (0%) |
| Total | 1,203  (100%) | 423  (100%) | 147  (100%) | 11  (100%) | 225  (100%) | 1  (100%) |

**Supplementary Table S4**. Sporozoite Rates and Annual EIR by species and malaria risk strata. Note: DC = District Council; CC = City Council; SR = Sprozoite Rate; EIR = Entomological Inoculation Rate; pos = positive

| **Malaria risk strata** | **Council** | **All species** | | ***An. gambiae* s.l** | | ***An. funestus* s.l** | |
| --- | --- | --- | --- | --- | --- | --- | --- |
|  |  | **SR in % (pos /tested)** | **Annual EIR** | **SR (%)**  **(pos/tested)** | **Annual EIR** | **SR (%)**  **(pos/tested)** | **Annual EIR** |
| **High risk** | Handeni DC  (Intervention) | 8.8  (114 / 1,291) | 33.0 | 9.3  (112/1,203) | 33.4 | 2.3  (2/88) | 0.3 |
|  | Muheza DC  (Control) | 2.1  (57/2,719) | 72.2 | 1.4  (6/423) | 7.4 | 2.2  (51/2,296) | 63.7 |
| **Moderate risk** | Tanga CC  (Intervention) | 2.1  (10/479) | 2.5 | 0.0  (0/147) | 0.0 | 3.0  (10/332) | 2.1 |
|  | Pangani DC  (Control) | 0.4  (1/245) | 1.3 | 0.0  (0/11) | 0.0 | 0.4  (1/234) | 1.0. |
| **Low risk** | Lushoto DC  (Intervention) | 0.9  (2/232) | 0.3 | 0.9  (2/225) | 0.3 | 0.0  (0/7) | 0.0 |
|  | Bumbuli DC  (Control) | 5.1  (2/39) | 7.4 | 0.0  (0/1) | 0.0 | 5.3  (2/38) | 3.0 |
